# “We don’t separate out these things. Everything is related”: Partnerships with Indigenous Communities to Design, Implement, and Evaluate Multilevel Interventions to Reduce Health Disparities

**DOI:** 10.1007/s11121-024-01668-9

**Published:** 2024-04-10

**Authors:** Elizabeth Rink, Sarah A. Stotz, Michelle Johnson-Jennings, Kimberly Huyser, Katie Collins, Spero M. Manson, Seth A. Berkowitz, Luciana Hebert, Carmen Byker Shanks, Kelli Begay, Teresa Hicks, Michelle Dennison, Luohua Jiang, Paula Firemoon, Olivia Johnson, Mike Anastario, Adriann Ricker, Ramey GrowingThunder, Julie Baldwin

**Affiliations:** 1 Department of Health and Human Development, Montana State University, 312 Herrick Hall, Bozeman, MT 59715, USA; 2 Department of Food Science and Human Nutrition, Colorado State University, 502 West Lake Street, Fort Collins, CO 80526, USA; 3 Division of Indigenous Environmental Health and Land-Based Healing, Indigenous Wellness Research Institute, University of Washington, Gergerding Hall GBO, Box 351202, Seattle, WA, USA; 4 Department of Sociology, Research, and Development/CIEDAR Center, COVID-19 Indigenous Engagement, University of British Columbia, 310-6251 Cecil Green Park Road, Vancouver, BC V6T 1Z1, Canada; 5 CIEDAR co-Lead. Department of Psychology, University of Saskatchewan, 9 Campus Drive, 154 Arts, Saskatoon, SK S7N 5A5, Canada; 6 Colorado School of Public Health, Centers for American Indian and Alaska Native Health, University of Colorado Anschutz Medical Campus, 13055 East 17th Avenue, Aurora, CO 80045, USA; 7 Division of General Medicine and Clinical Epidemiology, Department of Medicine, University of North Carolina at Chapel Hill School of Medicine, Chapel Hill, NC; Cecil G. Sheps Center for Health Services Research, University of North Carolina at Chapel Hill, 725 M.L.K. Jr Blvd, Chapel Hill, NC 27516, USA; 8 Institute for Research and Education to Advance Community Health (IREACH), Elson S. Floyd College of Medicine, Washington State University, 1100 Olive Way #1200, Seattle, WA 98101, USA; 9 Gretchen Swanson Center for Nutrition, 14301 FNB Pkwy #100, Omaha, NE 68154, USA; 10 Maven Collective Consulting, LLC, 15712 N Pennsylvania Avenue Cube 5, Edmond, OK 73013, USA; 11 Teresa Hicks Consulting, 1107 East Babcock Street, Bozeman, MT 59715, USA; 12 Oklahoma City Indian Clinic, 4913 W Reno Ave, 856 Health Sciences Quad, Suite 3400, Oklahoma City, OK 73127, USA; 13 Department of Epidemiology and Biostatistics; UCI Health Sciences Complex, University of California Irvine, Program in Public Health, 856 Health Sciences Quad, Suite 3400, Irvine, CA 92617, USA; 14 Fort Peck Community College, 605 Indian Ave.,, Poplar, MT 59255, USA; 15 Center for Health Equity Research, Northern Arizona University, P.O. Box 4065, Suite 120, Flagstaff, AZ 86011-4065, USA; 16 Fort Peck Tribal Health Department, 501 Medicine Bear Road, Poplar, MT 59255, USA; 17 Fort Peck Tribes Language and Culture Department, 603 Court Ave., Poplar, MT 59255, USA

**Keywords:** Multilevel intervention, Indigenous, Native American, Community advisory board, Partnerships, Design, Implementation, Evaluation

## Abstract

Multilevel interventions (MLIs) are appropriate to reduce health disparities among Indigenous peoples because of their ability to address these communities’ diverse histories, dynamics, cultures, politics, and environments. Intervention science has highlighted the importance of context-sensitive MLIs in Indigenous communities that can prioritize Indigenous and local knowledge systems and emphasize the collective versus the individual. This paradigm shift away from individual-level focus interventions to community-level focus interventions underscores the need for community engagement and diverse partnerships in MLI design, implementation, and evaluation. In this paper, we discuss three case studies addressing how Indigenous partners collaborated with researchers in each stage of the design, implementation, and evaluation of MLIs to reduce health disparities impacting their communities. We highlight the following: (1) collaborations with multiple, diverse tribal partners to carry out MLIs which require iterative, consistent conversations over time; (2) inclusion of qualitative and Indigenous research methods in MLIs as a way to honor Indigenous and local knowledge systems as well as a way to understand a health disparity phenomenon in a community; and (3) relationship building, maintenance, and mutual respect among MLI partners to reconcile past research abuses, prevent extractive research practices, decolonize research processes, and generate co-created knowledge between Indigenous and academic communities.

## Introduction

Indigenous peoples experience some of the greatest health inequities in North America (Centers for Disease Control and Prevention (CDC), 2013). Individual education strategies and patient-centered medical models do not fully address the complex layers contributing to health disparities among Indigenous peoples. Multilevel interventions (MLIs) are viewed as an intervention design appropriate to reduce health disparities among Indigenous peoples because they can address Indigenous communities’ diverse histories, cultures, worldviews, dynamics, politics, and environments (Jernigan et al., 2020; Johnson-Jennings et al., 2023; Manson, 2022). Indigenous intervention science highlights the importance of context-sensitive MLIs that focus on tribal concepts of the collective, not individuals, and prioritize Indigenous and local knowledge systems over western science methods (Trickett & Espino, 2004; Trickett et al., 2011).

This paradigm shift away from an individual-level focus to community-level focus underscores tribal engagement to integrate cultural and local belief systems, Indigenous Research Methods (IRM), qualitative and quantitative methods, and historical and political contexts into the creation of MLIs with Indigenous communities (Rasmus et al., 2020). The use of multiple methodologies with Indigenous-centered MLIs to reduce health disparities tells the story of what is actually “going on” by providing multidimensional perspectives to a health issue in a tribal community (Zuberi & Bonilla-Silva, 2008). This holistic approach highlights the value of the interconnectedness of all things and underscores the centrality of the partnerships between Indigenous community members and researchers to generate MLIs that are relevant and applicable for Indigenous peoples.

In this paper, three case studies of how Indigenous partners and researchers collaborated on stages of research in MLIs, including design, implementation, and evaluation, are presented. Our case studies support the attention to community engagement as foundational to the creation, generation, and interpretation of Indigenous-centered interventions (Table 1) (Allen, 2014). We discuss the following: (1) design of the Ya’a De land-based healing camp to reconnect and revitalize traditional land-based practices; (2) implementation of a multilevel diabetes nutrition education program and food security resource for American Indian and Alaska Native (AI/AN) adults living with type 2 diabetes (T2D); and (3) evaluation of Nen ŨnkUmbi/EdaHiYedo (We Are Here Now), a MLI to prevent sexual and reproductive health (SRH) disparities among American Indian (AI) youth.

## Methods

We used a constructivist case study approach to describe how Indigenous community members and researchers collaborated in one of three research stages (design, implementation, and evaluation). Our case study approach with an interpretive/social constructivist paradigm suggests that people are continuously developing meanings and understandings in social, cultural, and historical contexts (Baxter & Jack, 2008). It allows researchers to study complex phenomena within their contexts to develop interventions by providing a rich description to understand the multifaceted issues of community-engaged, multilevel approaches to health interventions for Indigenous peoples (Stake, 1995). Our units of analysis were three cases, bound by the length of time from the start of each project until the write-up of the present manuscript (DeMarrais & Lapan, 2004). We assessed, processed, and explained within culturally situated meanings and concrete details how diverse community-academic partnerships were constructed and leveraged during different research stages (Mortari, 2015). The three case studies were in separate phases of complementation and able to present variation in how community engagement was applied in MLIs. Rink, Stotz, and Johnson-Jennings took the lead to write their respective case studies based on discussions during a series of online meetings between December 2022 and March 2023. Rink, Stotz, and Johnson-Jennings reported to their research teams and community partners the manuscript’s content and progress. Manuscript drafts were shared with co-authors for review and feedback prior to submission.

## Case Studies

### Case Study #1: Design—The Ya’a De Land-Based Healing Camp

As a result of colonial and ongoing traumas, 4.9% of First Nations in Canada experience some of the highest rates of mental health distress (Statistics Canada, 2011). Indigenous communities have identified disconnection to original land-based practices and medicines as a primary barrier to healing and well-being. Increased connection to culture has been related to Indigenous persons’ increased well-being, particularly when integrated with land-based healing (Johnson-Jennings & Walters, 2023; Johnson-Jennings et al., 2020). Though land-based healing has occurred for centuries within Indigenous communities as a methodology, only recently has it begun to be used as a research method and health intervention. Land-based healing has been defined as purposefully centering healing upon the land and supplementing with western approaches (Johnson-Jennings et al., 2020), which inherently considers multiple levels of influence by focusing on the bidirectional influence of environment, or land, on human health. At the same time, land-based MLIs are complex and not easily implemented without a strong foundation of partnership and a shared vision.

The Ya’a De land-based healing camp pilot was driven by community needs and many years of planning. The healing camp objectives were to: (1) increase moderate to vigorous land use for health; (2) increase healthy coping mechanisms for mental health/well-being through reconnection to place; and (3) increase healthy behavioral changes related to the land for dealing with stress, including Denè-specific land-based healing practices. The initial pilot was co-developed based on the Indigenist Community Engaged, Land-Based Healing Approach as an overall framework for considering behavioral changes (Johnson-Jennings & Walters, 2023). This pilot also served as an additional step of research partnership building and established how the researchers and community partners could engage upon the land in a good way with a shared vision for health. This pilot study was completed in 2022 with results from qualitative pre- and post-semi-structured interviews currently being prepared for community dissemination in order to receive feedback and co-develop a multilevel, community-based, land-based healing intervention. Our case study illustrates the complex process of partnering, planning, and co-designing a multilevel project prior to research implementation, a process that is often missing from the literature.

The Ya’a De land-based healing camp partnership was initiated by the community through an interconnected network of kinships and happenstances. Several years ago, the Denè elder involved in the project was motivated by other ways of knowing (e.g., dreams, prayers, and hopes for his relatives and self) (Cajete, 2014) to develop land-based healing on his ancestral lands and sacred waters. During the elder’s search for assistance, he developed a relationship with a white ally who is a local community educator, and together they began identifying the project needs. These discussions eventually led to Johnson-Jennings being introduced to the community members via Zoom in Fall 2018. The community and researchers immediately began to share stories and establish kinship that solidified their partnership. As can be seen within an Indigenous social network, formal means of introduction and in-person visits are not always needed to connect the researcher and the community; however, the researcher’s openness to introductions and flexibility are key.

As the team grew, they implemented several strategies to build culturally sensitive research (Burnette et al., 2014). The first step included employing culturally appropriate, reflective listening and storytelling (Archibald & Xiiem, 2018), with Johnson-Jennings via Zoom, to continue sharing about the needs, desires, and hopes as related to the individual, community, ancestral, and land needs. Also needed for culturally sensitive research, both parties engaged in openness and transparency in discussing whether they had the resources and time to move forward in building a research relationship. They further embraced the principles of reciprocity, honesty, and openness throughout their iterative process. Johnson-Jennings also held fast to cultural humility (Foronda, 2020). Despite being Indigenous and holding similar worldviews and values, Johnson-Jennings recognized she was from a different Indigenous Nation, did not live on their territory, and had received extensive western academic indoctrination. The community partners appreciated this awareness and decided to move forward with the partnership. They clearly indicated the multiple levels that they wished to approach, which included the land, more than human kin, and ancestral connections. This example of partnership building illustrates the need for active self-reflection, listening, and checking in with the community members at all stages of their relationship, despite the researcher’s cultural identity.

During the COVID-19 pandemic, Johnson-Jennings continued to lay the groundwork for a potential MLI research project. She immersed herself in becoming more educated about the Denè community and history. Her time and financial resources towards the future pilot reflected her belief in the project and its potential impact on the health of the community. Her actions motivated the project partners to establish the Tachè Healing Waters Society. Over time, the partners realized that pandemic issues exacerbated Indigenous community mental health needs (Arriagada et al., 2020; Huyser et al., 2022). Motivated to act, project partners sought additional research infrastructure and partners, engaging the Covid-19 Indigenous Engagement Development and Research (CIEDAR) team to determine fit. The Indigenous CIEDAR Leads readily agreed to co-develop and co-implement the pilot.

The Elder Lockhart, collaborator on the project, emphasized the need for reconciliation and involvement of non-Indigenous allies. In Canada, the governmental Truth and Reconciliation Commission Principles emphasize working jointly together to establish and maintain a respectful living framework between Indigenous and non-Indigenous persons (The Justice Institute British Columbia, 2023). The elder explained that for land-based healing to continue to occur, we must work together to create a space in which we can engage in respectful living. Community allies, therefore, were welcomed to assist the community in their land-based endeavors, as long as they received culturally appropriate training for respectful engagement.

Hence, this allied partnership and respectful engagement became a goal within the Ya’a De pilot project. By being on the land together, the Indigenous and non-Indigenous academics and community members were able to develop a shared vision for health that was reflective of the community’s cultural beliefs, values, and interests. The Ya’a De project demonstrates that, especially within land-based healing, allocating time to build partnerships among Indigenous and non-Indigenous academics and community members to understand the land and the community’s kin/culture supports the generative process of co-creating a shared vision for a MLI land-based healing project (Johnson-Jennings et al., 2023).

### Case Study #2: Implementation—Exploring Food Insecurity and Pilot Testing a Diabetes Nutrition Education Program and Food Security Resource for Indigenous Adults Living with Type 2 Diabetes

As supported by the Social Ecological Framework, a person’s health is influenced by individual factors, such as health-related knowledge and behavior, as well as factors at the interpersonal, organizational, community, and policy levels (McLeroy et al., 1988). The National Institutes of Minority Health and Health Disparities framework also supports multilevel factors to health, and more specifically, Manson and colleagues developed a multilevel framework indicating the levels and layers of factors that influence Indigenous peoples’ health (Manson, 2022). Further, US Healthy People 2030 has put new emphasis on a key objective of addressing social determinants of health through multilevel and multi-sector interventions. The social determinants of health are described as the conditions where people are born, live, learn, work, play, worship, and age (Office of Disease Prevention & Health Promotion, 2019), and for Indigenous peoples, this also includes addressing implications of colonization and systemic racism (Satterfield & Frank, 2016). Health education for people with T2D has traditionally included individual-level education by members of a health care team. However, a key shift away from solely individual-level education has encouraged health educators to consider additional levels of influence (e.g., the food environment) that impact a person’s ability to prevent and/or manage a chronic disease (Tagtow et al., 2021).

Traditional Indigenous food systems have been devastated by systemic inequities and anti-Indigenous racism (Greenwood et al., 2018; Warne & Wescott, 2019). Consequently, AI/ANs are almost 2 times more likely to have T2D and 2.5 times more likely to die from T2D than non-Hispanic white adults (CDC, 2013). A healthy diet is key to managing T2D; however, AI/ANs often lack access to healthy food and disproportionately experience food insecurity (Pardilla et al., 2014). Food insecurity, defined as lack of consistent access to enough food for an active, healthy life (USDA Economic Research Service, 2018), negatively impacts one’s ability to manage blood sugar via multiple pathways (Gucciardi et al., 2014; Seligman & Berkowitz, 2019). AI/ANs experience food insecurity due to poverty, transportation barriers, and pervasive food deserts (Tarasuk et al., 2015). Among AI/ANs, MLIs to address food insecurity include expanding healthy food sections of small grocery stores and supporting local farmers and community gardens. Much of this work focuses on AI/ANs dwelling in rural and reservation settings (Gittelsohn & Trude, 2017; Jernigan et al., 2012; Satterfield & Frank, 2016), although 70% of AI/ANs live in urban areas (Indian Health Service, 2018). Despite AI/ANs profound T2D health disparities, we do not know if adding a food security resource to diabetes nutrition education could further improve T2D outcomes for AI/ANs who live in urban areas. We also do not know if adding a food security resource alone can improve T2D outcomes, nor do we know what type of food security resources are preferred, sustainable, and relevant for AI/ANs with T2D.

MLIs to address T2D health disparities among AI/ANs are needed to answer these questions (Stotz, Brega et al., 2021a, Stotz, McNealy et al., 2021b). Our project, “Exploring food insecurity and pilot testing a diabetes nutrition education program and food security resource for Indigenous adults living with type 2 diabetes,” is an MLI aimed at increasing access to culturally relevant nutrition education and reducing food insecurity for AI/AN adults with T2D. The study is a three-arm randomized controlled trial with groups: (1) diabetes nutrition education + food security resource; (2) diabetes nutrition education only; and (3) food security resource only. It is a 3-month intervention with 5 data collection timepoints: baseline, 1, 3, 6, and 9 months. Outcomes include the following: HbA1c, blood pressure, dietary intake, nutrition self-efficacy and knowledge, and food security among others. The intervention is taking place at one urban Indian health center—and key clinic partners are American Indian registered dietitian nutritionists (RDNs). The diabetes nutrition education curriculum was developed in collaboration with AI/AN partners, born from a partnership with the Shakopee Mdewakanton Sioux Community of Minnesota and the American Diabetes Association. Details as to how AI/AN community members, AI/AN-serving health care personnel, and Tribal leaders informed all phases of this program, entitled “What Can I Eat? Diabetes Nutrition Education for AI/AN with Type 2 Diabetes,” are published elsewhere (Stotz et al., 2022, Stotz, Brega et al., 2021a, Stotz, McNealy et al., 2021b).

To ensure the study’s cultural centeredness, the project engages a community advisory board (CAB) to provide guidance on all stages of the MLI throughout the 3-year project. The CAB provides guidance on recruitment and retention strategies, how to prioritize traditional Indigenous foods, interpretation of findings, and guidance on dissemination of findings. The CAB includes AI/AN people living with T2D (e.g., people who use the collaborating urban Indian clinic for their health care), their family members, experts on food security, and both western and traditional health care providers who were selected through a formal application process. The CAB is led by a trained, American Indian RDN with expertise in food insecurity and diabetes, who serves as a liaison between the research team and CAB. The location, frequency, and duration of each CAB meeting are determined by the CAB members and CAB leader. The CAB leader manages meeting agendas, minutes, and share-back with the research team. As a key effort in honoring knowledge, time, and wisdom, the CAB members are paid for their time. Additionally, the collaborating Indian health center is also funded through a subaward to compensate leadership for strategic planning, coordination, and staff time for teaching and data collection. The CAB began meeting in June 2023 and will continue to meet throughout the RCT intervention, which began in September 2023 and will run through late 2024.

As a key first decision, the 8-member CAB provided guidance on determining the food security resource that would increase sustainable community-based capacity between multilevel and multiple sectors (e.g., clinic, food retail, food growers). Examples of the food security resource could have included, but were not limited to, produce prescription program (e.g., grocery store gift cards to support the purchase of fresh produce, farm-to-clinic produce supply arrangement), transportation vouchers to assist with transit to grocery stores, or medically tailored meals or groceries. Provision of resources is most effective when tailored to the specific needs of the priority community and the specific resource landscape in which the intervention takes place (Rosas et al., 2016). Therefore, instead of proscriptively deciding which food security resources will be offered, researchers employed community-based participatory research (CBPR) principles of equal engagement to inform decision-making regarding key aspects of program design, implementation, evaluation, and sustainability. Ultimately, the CAB decided on unrestricted grocery store gift cards for use at a local grocery store chain as the food security resource provided in this intervention. After deliberating on the pros and cons of all possible food security resources, CAB members decided to honor the decision-making and sovereignty of each adult participant in the study and allow each participant to select what they wanted to purchase with the gift card (vs. restricting the card to fresh produce only, for example). By allowing participants to determine what they needed to care for themselves and their family with any given weekly grocery gift card, the intervention further recognizes Indigenous values of holistic health—and allows participants’ to determine what is “health promoting” in their family each week.

The CAB also advises this study on best practices to sustain food security services after the intervention, engage Tribally-owned or -operated food retailers to build Indigenous community capacity across multi-sector organizations (e.g., food retail and clinic), evaluate program satisfaction and success from the perspective of the participants (patients), and consider and accommodate Indigenous values in program implementation and evaluation (e.g., environmental health, community-based health). This robust partnership with the CAB helps ensure that the intervention will best serve AI/ANs with T2D and foster sustainability of the intervention (Fleischhacker et al., 2012).

### Case Study #3: Evaluation—Nen ŨnkUmbi/EdaHiYedo (“We Are Here Now”)

Higher rates of teen birth, low birth weight, sexually transmitted infections, hepatitis C virus, and human immuno-deficiency virus are more prevalent among AI adolescents in comparison to other non-Indigenous adolescents in the United States (Bowes et al., 2018; de Ravello et al., 2014; Martin et al., 2018). Previous research has established the historical, social, cultural, economic, educational, and environmental determinants of sexual and reproductive health (SRH) in AI adolescents, indicating that individual characteristics alone do not influence AI adolescents’ SRH disparities (Markham et al., 2015; Rutman et al., 2008; Sarche & Spicer, 2008; Whitbeck et al., 2004). Addressing these multiple, complex factors inherent in AI adolescents’ human ecology warrant MLIs.

Nen ŨnkUmbi/EdaHiYedo (“We Are Here Now”/NE) tests the efficacy of an MLI to prevent SRH disparities among AI adolescents ages 14 to 18 years living on the Fort Peck Reservation in Northeastern Montana (herein referred to as Fort Peck). NE is grounded in a longstanding partnership between the Fort Peck Tribes and Montana State University that began in 2006. NE includes four levels: (Level 1) an adaptation of a school-based SRH curriculum called Native Stand, designed to address individual-level factors that lead to sexual risk behaviors; (Level 2) a family-level home-based curriculum tailored to increase communication between adult family members and youth about SRH topics; (Level 3) a cultural mentoring component at the community level in which AI youth receive traditional teachings about topics related to SRH; and (Level 4) a multi-sectoral network of organizations collaborate at the Fort Peck systems level to coordinate SRH services for AI youth. NE utilizes a CBPR framework, a stepped wedge design (SWD), and mixed methodologies. A 4-member CAB of Fort Peck tribal members provides guidance, insight, and recommendations for NE. NE began in April 2018 with the intervention period starting in May 2019 and had an original project end date of November 2022. However, the COVID-19 pandemic extended NE’s intervention period to November 2023 with a new project end date of November 2024 (Anastario et al., 2023).

NE was developed based on an exploratory study, pilot intervention, and a 6-month period of meetings between NE’s CAB and the tribal-university research team (Fort Peck Community College, Montana State University, Northern Arizona University) (Rink et al., 2022). Meeting discussions focused on ensuring: (1) the intervention was culturally grounded in the Fort Peck Tribes’ belief systems; (2) the intervention design adhered to the Fort Peck Tribal Executive Board’s request that the intervention be holistic and include all of the communities and organizations across Fort Peck; (3) the intervention met the rigors of a randomized control trial design; (4) the intervention had adequate sample size to test the efficacy of a MLI; (5) data collection methods and content were relevant to the tribal context and SRH research field; and (6) the story of NE could be shared with other tribal communities (Rink et al., 2022).

The evaluation of NE’s effectiveness as a MLI SRH intervention for AI youth included several components (Rink et al., 2022). A SWD trial was selected because it was consistent with tribal members’ desires that all 14- to 18-year-old AI youth receive the intervention. The SWD is a cluster-randomized trial, which involves random and sequential crossover of clusters from control to intervention until all clusters are exposed to the intervention (Brown & Lilford, 2006; Hemming et al., 2015; Hussey & Hughes, 2007; Mdege et al., 2011; Woertman et al., 2013). NE involved five schools each randomly assigned to its own sequence. Schools crossed over from control to intervention based on their sequence. Data were collected at four time points in each sequence, as noted below, resulting in an incomplete open-cohort SWD. It should be emphasized that NE’s final SWD trial emerged from a complex interplay of epistemologies and interests, where inputs from the CAB, tribal institutions, schools on the reservation, and western research priorities were considered.

We conducted a simulation-based analysis to determine NE’s statistical power. An intraclass correlation coefficient of 0.016 within schools, similar to that used in a HIV prevention study among AI adolescents, was employed (Kaufman et al., 2014). The temporal autocorrelation for individual-level consecutive observations in our initial data was 0.45. The school-level autocorrelation was 0.83. A zero-inflated Poisson model was simulated using a two-tier method. First, a Bernoulli model determined the proportion of sexually inactive participants (60%, as indicated by preliminary data). Then, for those deemed potentially active, we simulated a count of sexual partners using a Poisson distribution, considering temporal and within-school correlations as mentioned earlier. Our simulation also incorporated an open-cohort design, mirroring accrual and attrition rates at various time points as seen in the preliminary data. We altered the percentage decrease in the outcome to evaluate the power in detecting an effect of this magnitude. The simulation findings indicate that the SWD trial possesses an 80% probability of identifying a 34% reduction in the number of sexual partners among the participating youth (Anastario et al., 2023).

Quantitative data was collected using surveys for NE’s SWD trial. Student participation in NE’s Level 1 was assessed with a student survey administered in the 5 schools participating in NE at baseline, 3-month mid-intervention, post-intervention, and 3-month post-intervention. Parent participation in NE’s Level 2 was assessed with a parent/legal guardian survey administered in the home or at school at baseline, post-intervention, and 3-month post-intervention. NE’s Level 3 cultural component was assessed with measures that were included in the student and partner surveys.

In trial end analyses, we plan to use generalized linear mixed-effects models (GLMM) to model outcomes and to provide proper weighting when cluster sizes vary (Fitzmaurice et al., 2008; Hussey & Hughes, 2007). Since we did not collect data beyond the 3-month follow-up period based on recommendations from the CAB and the schools and tribal institutions partnering with NE (where the effect of NE is assumed to be more stable), our analyses will likely avoid potential misinterpretation of the trial’s efficacy had measurements persisted over a longer period of time (Kenny et al., 2022; Maleyeff et al., 2023). Further, based on our experience with NE’s implementation, adherence to MLI SWD protocols in tribal communities was complex given the contextual realities of student attrition, school schedules, participation in high school sports, adverse events within the tribal communities where NE’s participating schools were located, and study design adjustments due to the COVID-19 pandemic (Anastario et al., 2023; Rink et al., 2022). Because of these varying external factors, a future adaptation of NE may warrant a staircase cluster randomized trial design which allows for omitting multiple baseline measurement periods (Grantham et al., 2024).

NE’s Level 4 was assessed by monitoring the barriers, facilitators, and solutions of Fort Peck organizations ability to coordinate SRH services for AI youth at Fort Peck. NE’s Level 4 analysis has been conducted using reflexive inquiry to assess, process, and explain how SRH services for AI youth at Fort Peck are coordinated and accessed within a tribal context (Mortari, 2015).

In addition to NE’s SWD to test the efficacy of our intervention, NE’s fidelity and acceptability were evaluated using tracking logs developed to collect intervention dose and adherence to intervention protocols, use of intervention skills by research participants, and intervention acceptability and are completed in real time as the intervention was implemented in each cluster (school) (Bellg et al., 2004). Focus groups were an additional qualitative measure to assess fidelity and acceptability, given the utility of focus groups in inductively determining key issues and ideas, their ability to elucidate process-oriented outcomes, and their use in previous SRH intervention evaluation studies (Morgan, 1996; Naggy Hesse-Biber & Leavy, 2010). Six focus groups (2 youths, 2 parents, 2 professionals) with 6 to 8 individuals each were conducted at the end of each cluster completion. NE’s fidelity and acceptability are assessed qualitatively using content analysis to analyze the tracking logs and focus groups (Charmaz, 2014; Corbin & Strauss, 2008).

Iya Waste (“To Speak Good Words”) is the final phase included in the evaluation of NE’s efficacy, fidelity, and acceptability. Iya Waste is derived from Dakota/Nakota practices that teach others about the world around them in a good way through sharing experiences. Iya Waste was developed by the NE research team and CAB as an Indigenous Research Methodology (IRM) specific to the Dakota/Nakota Nations of the Fort Peck Tribes to describe the contextual, cultural, and political factors that were at play during NE’s implementation in each cluster and how the quantitative and qualitative data may be understood and relevant to NE’s implementation and overall findings. During quarterly meetings with the CAB and the tribal-university research team, quantitative and qualitative data is reviewed as necessary in an iterative participatory, reflective dialogue to center and prioritize the meaning of the data from a tribal perspective within the academic literature (Hallett et al., 2017; Jull et al., 2019; Kroik et al., 2020). NE’s baseline data and interim data were discussed and understood in such a way (Anastario et al., 2023; Rink et al., 2023). Iya Waste supports the emerging importance of integrating IRM into clinical trials by using narration to “tell the story” of what took place in a community during trial implementation to understand and contextualize implementation processes and outcomes.

## Discussion and Conclusion

**Fig. 1:**
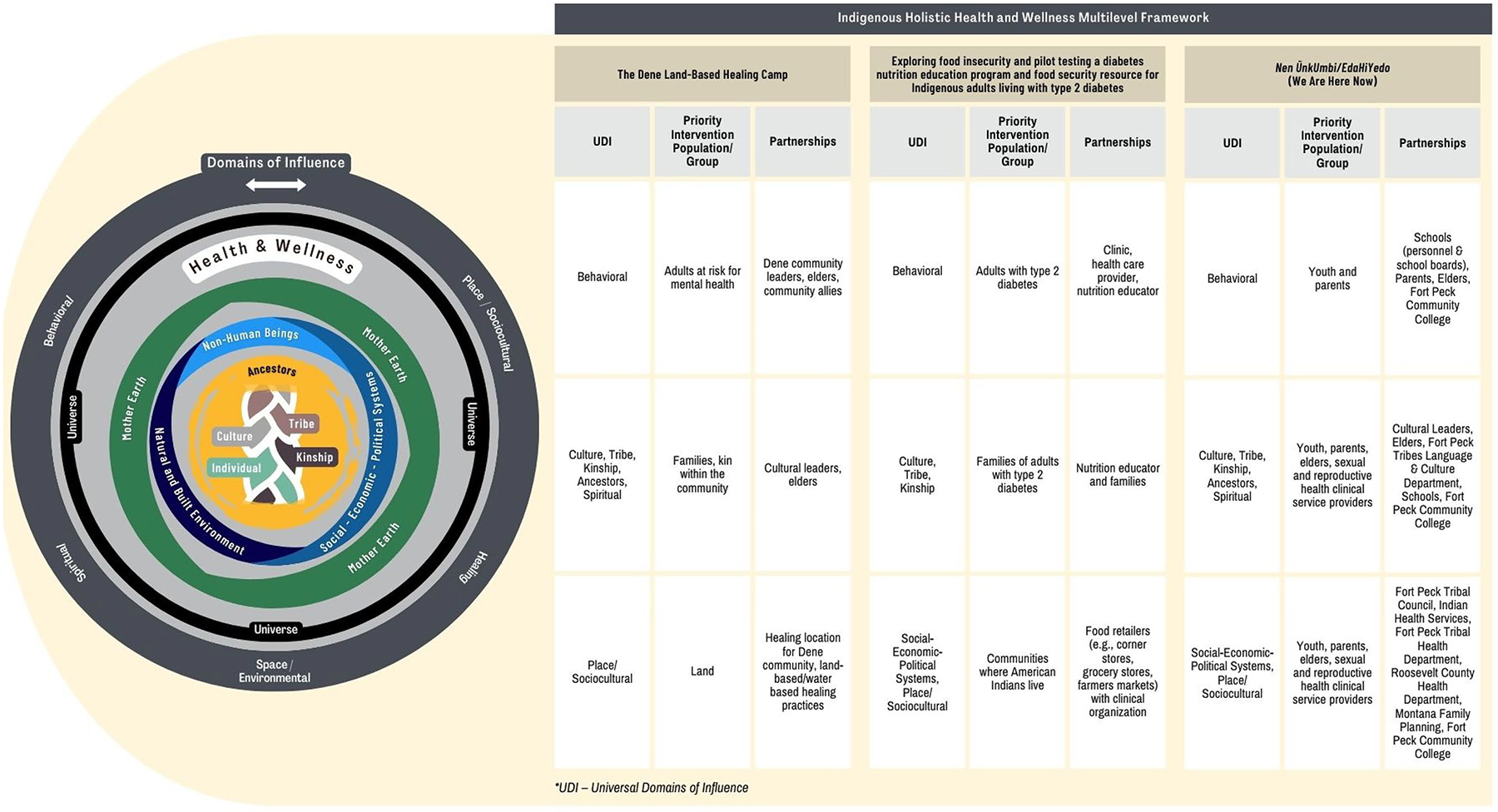
An overview of the partnerships in MLIs by building on the Indigenous Holistic Health and Wellness Multilevel Framework Note: Integration of the Indigenous Holistic Health and Wellness Framework to address universal domains of influence, priority populations, and partnerships. Reprint of the figure from “All systems are interrelated: Multilevel interventions with indigenous communities,” by Michelle Johnson-Jennings, Elizabeth Rink, Sarah Stotz, Maya Magarati, Roland Moore, 2023, *Contemporary Clinical Trials*, 124, p. 2.

While the impacts of tribal-academic partnerships on MLIs may differ across studies, they are also transferable and replicable for moving our understanding forward in how and with whom to engage with Indigenous communities to design, implement, and evaluate such complex, multifaceted studies. We provide an overview of how the partnerships in our respective MLIs were integrated into our studies by building on the Indigenous Holistic Health and Wellness Multilevel Framework developed by the authors and colleagues (Fig. 1) (Johnson-Jennings et al., 2023). Three key understandings from the case studies highlight a multilevel framework to engaging with tribal communities in different ways and at different times.

First, in the Ya’a De land-based healing camp and NE, researchers were approached by the community to conduct their studies, signaling the desire and commitment of the community to the research and the trust community members had in the researchers. Further, the researchers spent substantiative time collaborating with the community to ensure the intervention was in alignment with community values. For example, Johnson-Jennings met with key stake-holders and elders for 2 years to design the land-based healing camp, and Rink worked for 4 years to conduct exploratory research and a pilot test, which led to NE’s creation. These two examples speak to the importance of time spent with tribal communities to design complex interventions that require iterative, consistent conversations within research teams and with collaborators over time to synthesize the necessary components to form the multiple, diverse partnerships necessary for MLIs.

Second, conversations among tribal partnerships in the three case studies led to the inclusion of qualitative and/or Indigenous methods, thereby honoring Indigenous and local knowledge systems and ways of understanding a health disparity. Thus, the MLIs discussed here tell the story of how our different stages in the research process (design, implementation, evaluation) take into consideration the cultural, social, structural, and political dynamics to contextualize MLI outcomes.

Third, CBPR practices, such as co-learning and co-sharing, were inherent in our respective community-academic partnerships. Our partnerships included a wide range of relationships with individuals, groups, and organizations, such as elders, cultural leaders, CABs, schools, health clinics, food retailers, and the land. The relationship building, maintenance, and mutual respect required for ongoing partnerships necessary for MLIs contributes to the reconciliation of past abuse, prevents extractive research practices, decolonizes the research processes, and leads to the co-creation of new knowledge between Indigenous communities and their academic partners.

In conclusion, MLIs with Indigenous communities encompass a diverse set of partnerships to ensure their culture and context are accurately and respectfully reflected and integrated into the MLIs. The inclusion of diverse partnerships in Indigenous-centered MLIs assists in understanding how such complex multilevel studies can be designed, implemented, and evaluated with the integration of cultural values and worldviews to accurately reflect the cultural and contextual nuances necessary to address health disparities with Indigenous communities. Underlying the approaches discussed in our case studies is a response to decolonize the way in which MLIs are conducted with Indigenous communities. Our work exemplifies generative research practices developed as a result of our community partnerships that contribute to new ways of actualizing Indigenous-centered MLIs.

## Figures and Tables

**Table 1:** Community engagement in the design, implementation, and evaluation of multilevel interventions with Indigenous communities

DESIGN			
	Description	Method of Community Engagement	Impact on Multilevel Intervention
The Ya’a De Land Based Healing Camp	This project entails developing a land-based healing intervention in the Northwest Territories through collaborative engagement and curriculum development to improve mental health.	The community approached Johnson-Jennings, based on her expertise in land-based healing, and requested assistance in designing a land-based healing camp. Over the course of two years, Johnson-Jennings and the CIEDAR team met with the community key stakeholders and elder to learn of his vision for this project. They engaged the elder, key community stakeholders, and the land in their process of piloting a land-based healing camp.	• Integration of ancestral original instructions, elders, and land-based practices • Co-learning and co-sharing with Indigenous and non-lndigenous allies (reconciliation) • Bidirectional healing with the land • Consideration of the impact on environment, land, and human health • The process of how to engage in multi-level interventions involvinq land-based healing
IMPLEMENTATION			
	Description	Method of Community Engagement	Impact on Multilevel Intervention
Exploring food insecurity and pilot testing a diabetes nutrition education program and food security resource for Indigenous adults living with type 2 diabetes	This project includes implementing a diabetes nutrition education curriculum at one urban Indian clinic for adults diagnosed with type 2 diabetes. Two of the three arms of the randomized controlled study will also receive an added healthy food access resource to understand if nutrition education, nutrition education + healthy food resource, or healthy food resource alone has benefits on diabetes self-management and care.	This project includes a robust 8-person community advisory board led by an American Indian registered dietitian nutritionist. The community advisory board informs all stages of the project as a key means to promote long term sustainability of the project, should the community be interested in continuing after the evaluation is complete.	• Integration of quantitative, qualitative, and Indigenous Research methods • Collaboration and communication between community advisory board and university-based research team in determining how to best sustain the clinic and food retail relationship • Community advisory board includes members from local food retail outlets (e.g., store owners, fanners), clinic representatives, and participants to explore best practices for building local cross sector capacity.
EVALUATION			
	Description	Method of Community Engagement	Impact on Multilevel Intervention
*Nen UnkUmbi/EdaHiYedo* (We Are Here Now)	This project aims to reduce sexual and reproductive health disparities among 14–18-year-old Al youth. There are four levels of intervention (individual, family, community, systems). We use a Stepped Wedge Design within a CBPR framework and qualitative and quantitative methods as well as Indigenous Research Methods.	The evaluation was designed with the tribal-university research team and community advisory board to include quantitative and qualitative methods, *lya Waste* (“to speak good words”) was also used for evaluation. The community advisory board and tribal-university research team engage in iterative participatory reflective to discuss the meaning of the quantitative and qualitative data within the social, cultural, and political context of the Fort Peck reservation in order to tell the story of NE.	• Integration of quantitative, qualitative, and Indigenous Research Methods • Co-leaming and co-sharing between community advisory board and tribal-university research team in determining how contextual factors influence NE’s implementation and outcomes Tells the story of how NE was implemented and identifies events and situations relevant to trial implementation in addition to analysis of outcome variables
